# Supplementary figures and images for: Loss-of-function of Fbxo10, encoding a post-translational regulator of BCL2 in lymphomas, has no discernible effect on BCL2 or B lymphocyte accumulation in mice
Source: PLoS One. 2021 Apr 29;16(4):e0237830. doi: 10.1371/journal.pone.0237830 (PMC8084200; doi:10.1371/journal.pone.0237830)

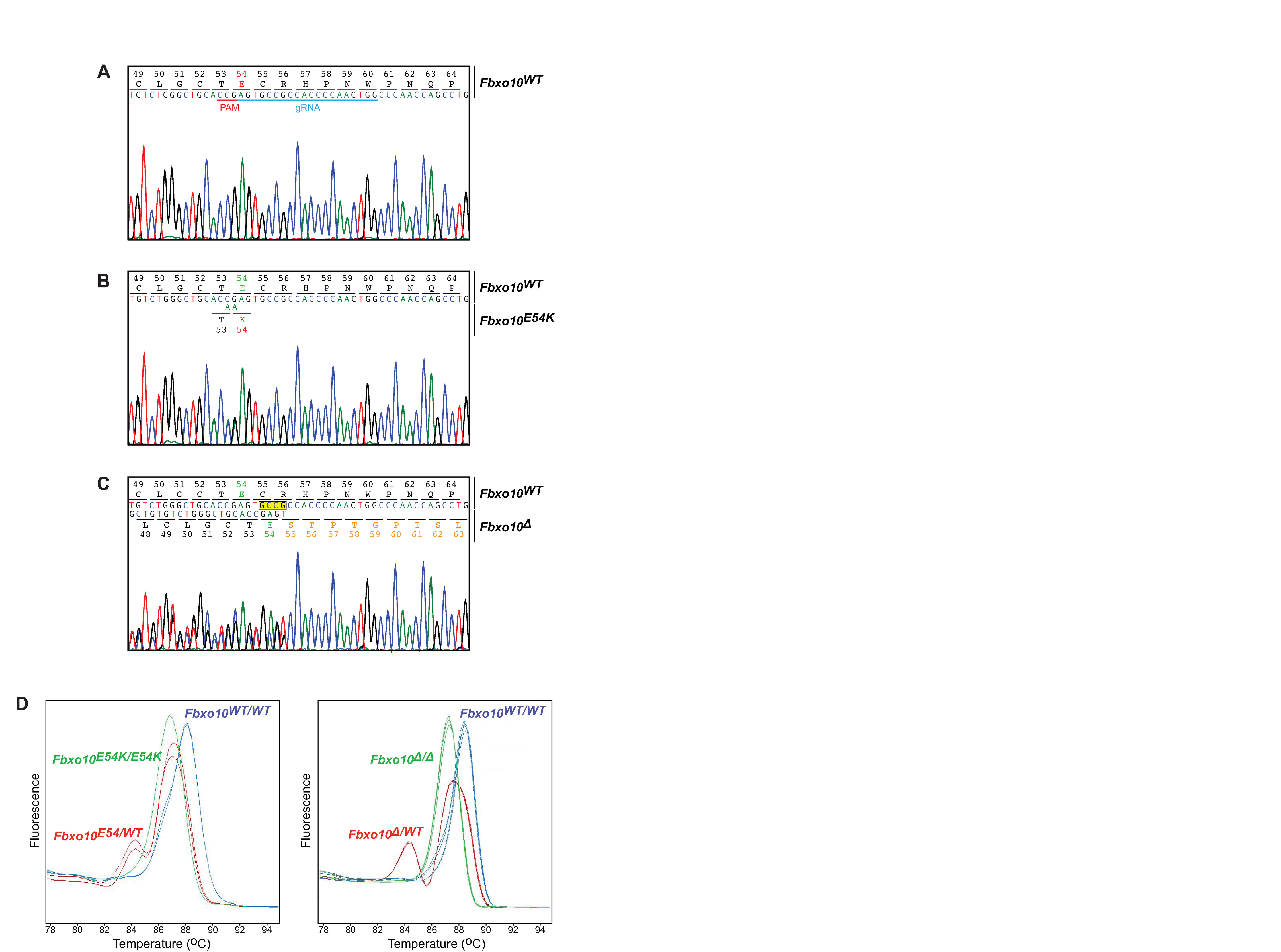

Supplement: S1 Fig — CRISPR/Cas9-engineered mice were produced as described in the materials and methods. (A-C) Sanger sequencing traces of a portion of the Fbxo10 gene encoding amino acids 49 to 64 of FBXO10: (A) wild type sequence showing the target site of the sgRNA used and its associated PAM and highlighting the E54 codon (B) sequence from a mouse heterozygous for the E54K mutation (together with the PAM inactivating mutation in the T53 codon) (C) sequence from a mouse heterozygous for a four base pair deletion (GCCG, highlighted) resulting in a reading frame shift after the E54 codon. Note that the Sanger sequencing read is from right to left, and the two frameshifted sequences that are superimposed downstream of the deletion are shown above. (D) High resolution melt curve analysis plots of DNA from wild-type and mutant mice, showing the alternative fluorescence profiles resulting from heterozygous or homozygous E54K (left) or C55SfsTer55 (right) mutations. (TIF) [file pone.0237830.s001.tif]

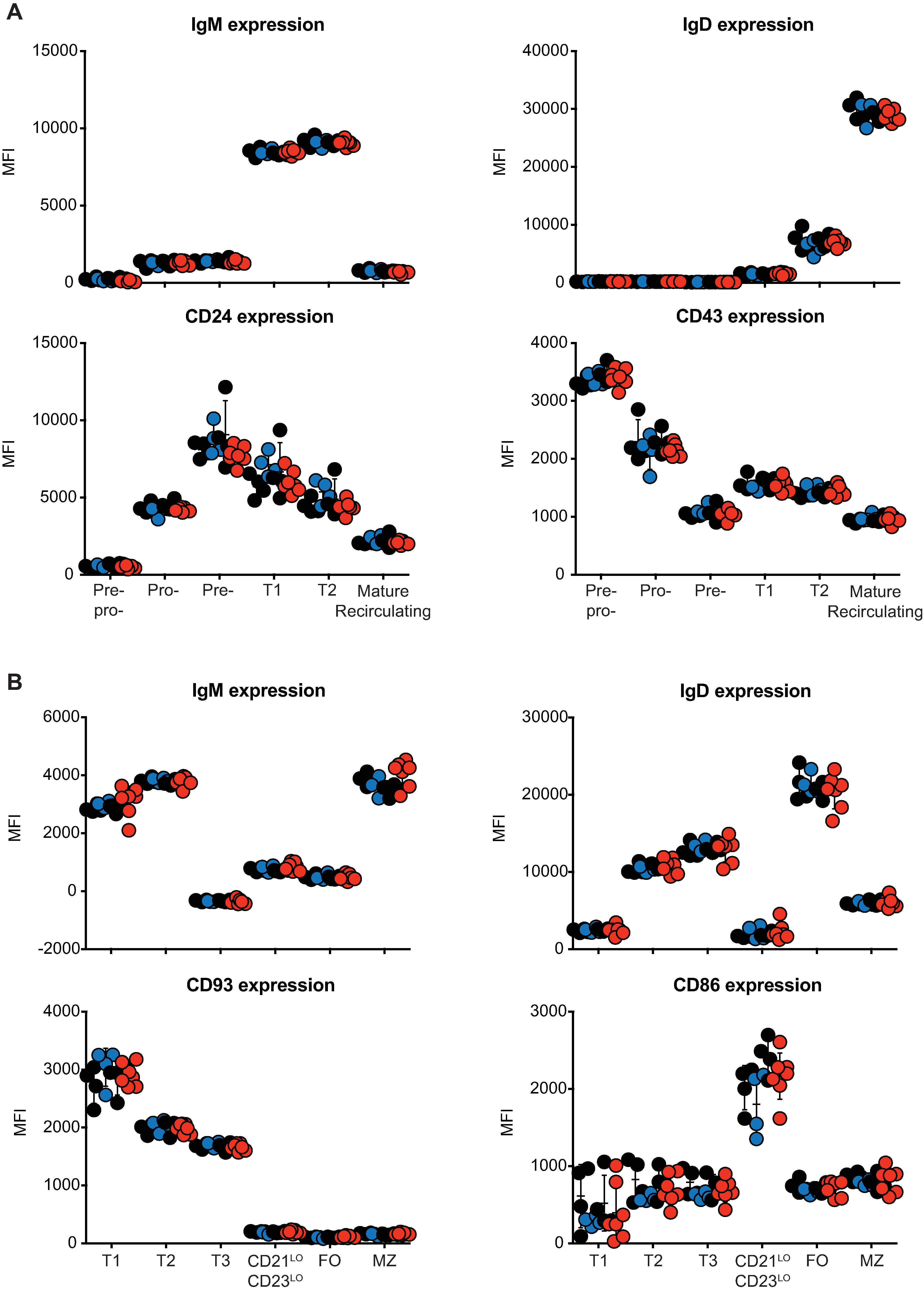

Supplement: S2 Fig — (A) Mean fluorescence intensity (MFI) for IgM, IgD, CD93, CD86 expression in splenic B cell subsets of Fbxo10+/+, Fbxo10fs/fs, Fbxo10E54K/E54K mice 40–50 weeks old. (B) MFI for IgM, IgD, CD24, CD43 expression in bone marrow B cell subsets of Fbxo10+/+, Fbxo10fs/fs, Fbxo10E54K/E54K mice 40–50 weeks old. Each dot represents an individual biological replicate in Fbxo10+/+ (black), Fbxo10E54K/E54K (red) or Fbxo10fs/fs (blue) mice. Similar results were obtained for multiple protein markers (CD19, CD21/35, CD23, CD24, CD43, CD86, etc.). Results are representative of n = 2 experiments on un-immunised mice 40–50 weeks old and similar results were obtained for n = 2 experiments on mice 10–20 weeks old, 7 days post-immunisation with SRBC. Statistical analysis: t-test corrected for multiple comparisons using the Holm-Sidak method yielded no evidence for significant differences between mutants and wildtype controls with p < 0.05. (TIF) [file pone.0237830.s002.tif]

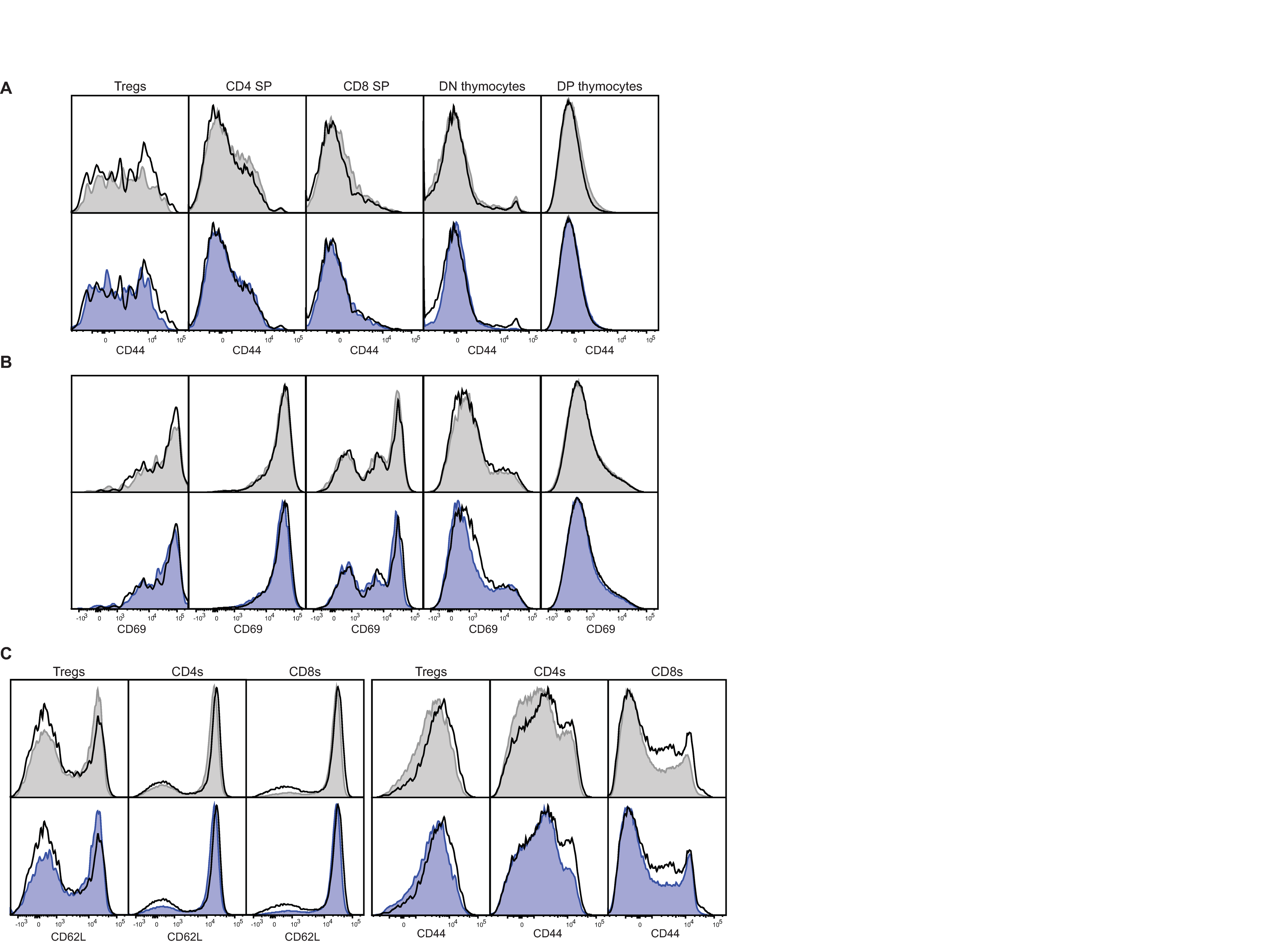

Supplement: S3 Fig — (A,B) Representative histogram overlays of Fbxo10+/+ (grey fill) or Fbxo10fs/fs (blue fill) thymocyte subsets relative to Fbxo10+/+ control thymocytes (black line) for CD44 (A) or for CD69 (B). Results are representative of results obtained for other markers: CD25, CD69, PD1, CD3, etc. (C) Representative histogram overlays of Fbxo10+/+ (grey fill) or Fbxo10fs/fs (blue fill) splenic T cells relative to Fbxo10+/+ control cells (black line) showing CD62L (left 3 panels) or CD44 (right 3 panels). Results are representative of results obtained for other markers: CD25, CD62L, PD1, CD3, etc. (TIF) [file pone.0237830.s003.tif]

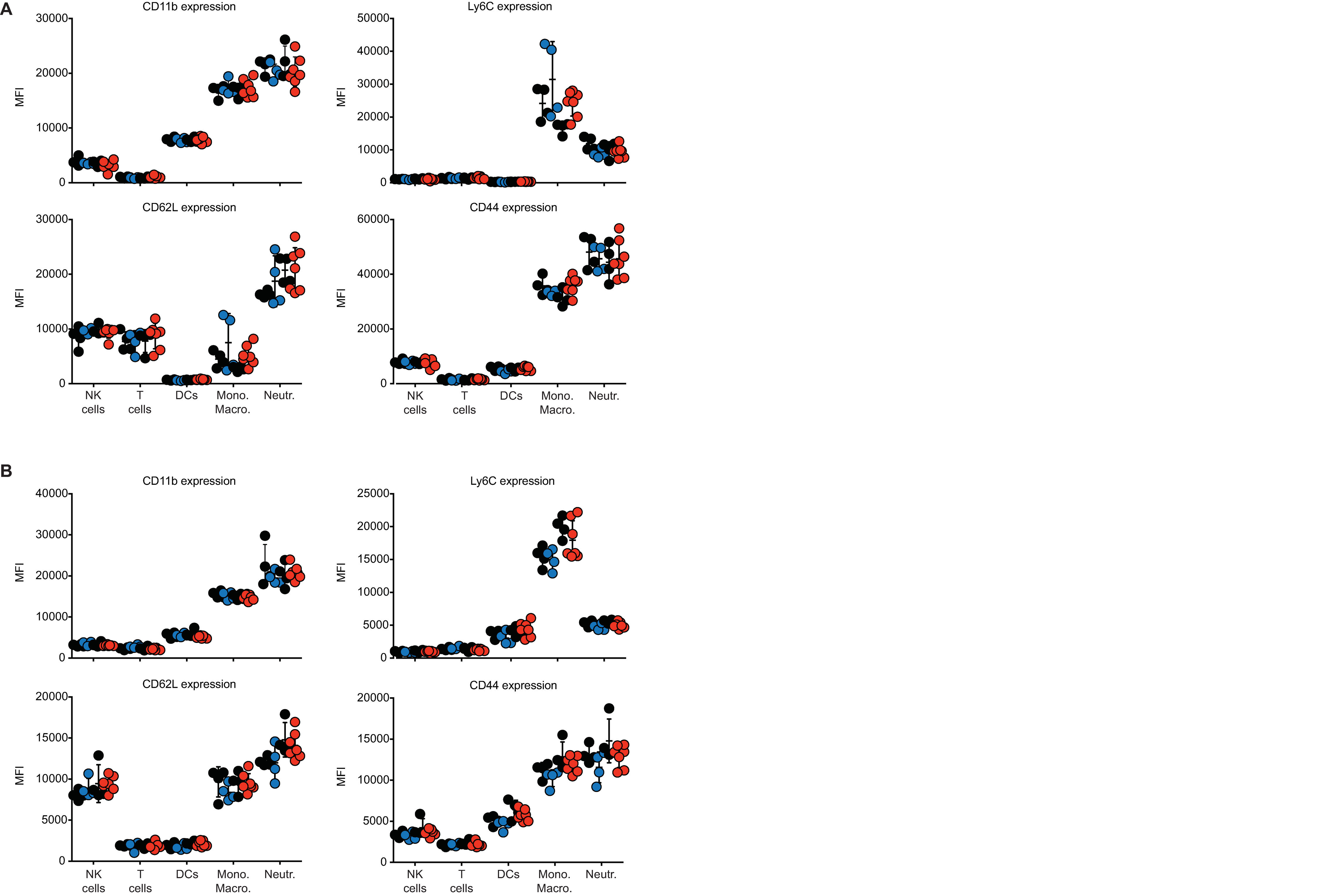

Supplement: S4 Fig — (A) Mean fluorescence intensity (MFI) for CD11b, Ly6G, CD62L, CD44 expression in spleen leukocyte subsets of Fbxo10+/+, Fbxo10fs/fs, Fbxo10E54K/E54K mice 40–50 weeks old. (B) Mean fluorescence intensity (MFI) for CD11b, Ly6G, CD62L, CD44 expression in bone marrow leukocyte subsets of Fbxo10+/+, Fbxo10fs/fs, Fbxo10E54K/E54K mice 40–50 weeks old. Each dot represents an individual biological replicate in Fbxo10+/+ (black), Fbxo10E54K/E54K (red) or Fbxo10fs/fs (blue) mice. Similar results were obtained for multiple protein markers (NK1.1, Ly6G, FSC, SSC-A, MHC II, etc.). Results are representative of n = 2 experiments on un-immunised mice 40–50 weeks old and similar results were obtained for n = 2 experiments on mice 10–20 weeks old, 7 days post-immunisation with SRBC. Statistical analysis: t-test corrected for multiple comparisons using the Holm-Sidak method yielded no evidence for significant differences between mutants and wildtype controls with p < 0.05. (TIF) [file pone.0237830.s004.tif]

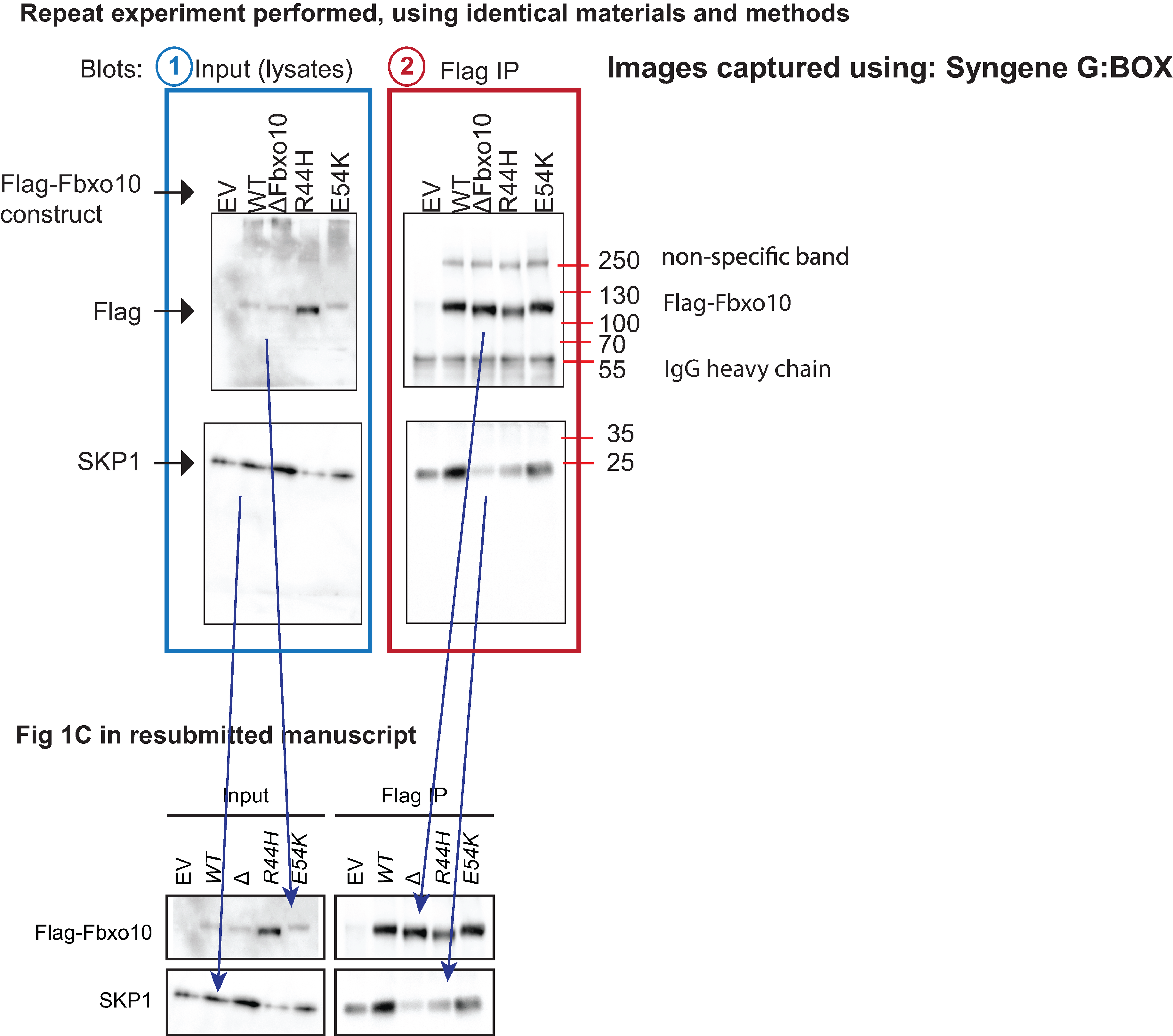

Supplement: S1 Raw images — (TIF) [file pone.0237830.s005.tif]
